# RETRACTED: Choi et al. PEP-1-GLRX1 Reduces Dopaminergic Neuronal Cell Loss by Modulating MAPK and Apoptosis Signaling in Parkinson’s Disease. *Molecules* 2021, *26*, 3329

**DOI:** 10.3390/molecules31142471

**Published:** 2026-07-15

**Authors:** Yeon Joo Choi, Dae Won Kim, Min Jea Shin, Hyeon Ji Yeo, Eun Ji Yeo, Lee Re Lee, Yejin Song, Duk-Soo Kim, Kyu Hyung Han, Jinseu Park, Keun Wook Lee, Jong Kook Park, Won Sik Eum, Soo Young Choi

**Affiliations:** 1 Department of Biomedical Science and Research Institute of Bioscience and Biotechnology, Hallym University, Chuncheon 24252, Republic of Korea; cyj0036@hallym.ac.kr (Y.J.C.); wehome3@hallym.ac.kr (M.J.S.); hj0428@hallym.ac.kr (H.J.Y.); ej428@hallym.ac.kr (E.J.Y.); 20173637@hallym.ac.kr (L.R.L.); khhan@hallym.ac.kr (K.H.H.); jinpark@hallym.ac.kr (J.P.); keunwook@hallym.ac.kr (K.W.L.); jkpark@hallym.ac.kr (J.K.P.); 2 Department of Biochemistry and Molecular Biology, Research Institute of Oral Sciences, College of Dentistry, Gangneung-Wonju National University, Gangneung 25457, Republic of Korea; kimdw@gwnu.ac.kr; 3 Department of Anatomy and BK21 FOUR Project, College of Medicine, Soonchunhyang University, Cheonan-si 31538, Republic of Korea; 20217050@sch.ac.kr (Y.S.); dskim@sch.ac.kr (D.-S.K.)

The journal retracts the article titled, “PEP-1-GLRX1 Reduces Dopaminergic Neuronal Cell Loss by Modulating MAPK and Apoptosis Signaling in Parkinson’s Disease” [1], cited above.

Following publication, concerns were brought to the attention of the Editorial Office regarding the integrity of the images presented in this publication [1].

In accordance with the journal’s standard procedures, the Editorial Office and Editorial Board conducted an investigation that confirmed concerns in Figure 8, including inappropriate image editing and duplication between panels presented in this figure and material from and earlier published article [2], presenting different experimental conditions. Although the authors cooperated with the investigation, the explanations and supporting materials provided were not deemed sufficient by the Editorial Board to resolve the identified concerns. As a result, the Editorial Board has lost confidence in the reliability of the findings and has decided to retract this publication [1], as per MDPI’s retraction policy.

This retraction was approved by the Editor-in-Chief of the journal *Molecules*.

The authors have been informed of this retraction but did not provide a comment on this decision.

## References

[1] Choi Y.J., Kim D.W., Shin M.J., Yeo H.J., Yeo E.J., Lee L.R., Song Y., Kim D.-S., Han K.H., Park J. (2021). RETRACTED: PEP-1-GLRX1 Reduces Dopaminergic Neuronal Cell Loss by Modulating MAPK and Apoptosis Signaling in Parkinson’s Disease. Molecules.

[2] Kim D.S., Sohn E.J., Kim D.W., Kim Y.N., Eom S., Yoon G.H., Cho S.W., Lee S.H., Hwang H.S., Cho Y.S. (2012). PEP-1-p18 prevents neuronal cell death by inhibiting oxidative stress and Bax expression. BMB Rep..

